# Syntactic chunking reveals a core syntactic representation of multi-digit numbers, which is generative and automatic

**DOI:** 10.1186/s41235-022-00409-2

**Published:** 2022-07-06

**Authors:** Dror Dotan, Nadin Brutmann

**Affiliations:** Mathematical Thinking Lab, School of Education and School of Neuroscience, Tel Aviv University, Tel Aviv, Israel

**Keywords:** Number syntax, Chunking, Symbolic numbers, Multi-digit number comprehension

## Abstract

Representing the base-10 structure of numbers is a challenging cognitive ability, unique to humans, but it is yet unknown how precisely this is done. Here, we examined whether and how literate adults represent a number’s full syntactic structure. In 5 experiments, participants repeated number-word sequences and we systematically varied the order of words within each sequence. Repetition on grammatical sequences (e.g., two hundred ninety-seven) was better than on non-grammatical ones (hundred seven two ninety). We conclude that the participants represented the number’s full syntactic structure and used it to merge number words into chunks in short-term memory. Accuracy monotonously improved for sequences with increasingly longer grammatical segments, up to a limit of ~ 4 words per segment, irrespectively of the number of digits, and worsened thereafter. Namely, short chunks improved memorization, whereas oversized chunks disrupted memorization. This chunk size limit suggests that the chunks are not based on predefined structures, whose size limit is not expected to be so low, but are created ad hoc by a generative process, such as the hierarchical syntactic representation hypothesized in Michael McCloskey’s number-processing model. Chunking occurred even when it disrupted performance, as in the oversized chunks, and even when external cues for chunking were controlled for or were removed. We conclude that the above generative process operates automatically rather than voluntarily. To date, this is the most detailed account of the core representation of the syntactic structure of numbers—a critical aspect of numerical literacy and of the ability to read and write numbers.

**Supplementary Information:**

The online version contains supplementary material available at 10.1186/s41235-022-00409-2.

## Significance statement

The ability to read and write numbers is a critical aspect of numerical literacy and a major predictor of elementary-school math achievements. An underappreciated fact is that reading and writing numbers is also very hard: Even literate adults make many errors in these tasks, and about 8% never become good at it and have dysnumeria, a prevailing learning disorder in number reading or writing. The central origin of these difficulties is the ability to handle the number’s syntactic structure, i.e., to combine digits or words into a multi-digit number or to decompose a multi-digit number into its elements. It is perhaps not surprising that syntax is the crux of the difficulty, as number syntax is hypothesized to reflect a more-general ability, which is cognitively demanding and may be unique to humans, to represent complex structured information in a recursive or hierarchical manner. Here, we examined in detail this syntactic processing. We show that literate adults can form a cognitive representation of the syntactic structure of a whole number, even for numbers as long as 6 digits, and to do so they use an automatic process (as opposed to applying a learned strategy) that creates the syntactic representation in a step-by-step manner (as opposed to just retrieving a predefined representation). These conclusions can help improve how we teach numbers in elementary school, and how we identify and treat individuals with dysnumeria.

## Introduction

Numerical literacy is extremely important in modern society. It is useful in everyday life, it is crucial for most academic and scientific disciplines, and it predicts academic achievements, unemployment, salaries, and mental and physical health (Duncan et al., 2007; Ritchie & Bates, 2013). There are many aspects to being proficient with numbers and in mathematics, and a central one is the ability to read and write numbers. In elementary school, this skill turns out to be a main predictor of arithmetic abilities (Habermann et al., 2020). Later in life, most educated adults can read and write numbers accurately and without difficulties, but a surprisingly large number of people find it quite hard even as adults. For example, a recent study examined 120 literate adults and found that 9 of them (7.5%) had considerable difficulties in reading multi-digit numbers—they erred in more than 14% of the numbers they were asked to read (Dotan & Handelsman, in prep.). These people are likely to satisfy the criteria for dysnumeria, a learning disorder that disrupts number reading (Dotan & Friedmann, 2018).

As it turns out, the difficulties in reading and writing numbers are not random but follow a consistent pattern, linking them to specific cognitive mechanisms of number processing. A central classification of the number-processing mechanisms is into lexical processes, which handle the identity of each digit or number word, and syntactic processes, which handle the relations among lexical items. For example, identifying a digit or retrieving a number word are lexical processes, whereas detecting how many digits a number has, and the decimal role of each digit, are syntactic processes (Cappelletti et al., 2005; Cipolotti, 1995; Cipolotti et al., 1994; Deloche & Willmes, 2000; Dotan & Friedmann, 2018; Furumoto, 2006; McCloskey et al., 1986; Noël & Seron, 1993). Among these two, it is syntax that poses the bigger challenge. Learning to process the syntax of numbers during childhood takes years to master and continues long after the lexical knowledge—the digits and the number-word names—was obtained (Cheung & Ansari, 2020; Dotan & Dehaene, 2016; Shalit & Dotan, 2022). Moreover, when reading numbers, children (Moura et al., 2013; Power & Dal Martello, 1990, 1997; Shalit & Dotan, 2022; Steiner et al., 2021) and adults (Dotan & Friedmann, 2018; Dotan & Handelsman, in prep.) make more syntactic than lexical errors. Finally, the main reason for dysnumeria, the learning disorder that disrupts number reading, is inability to process the number’s syntactic structure properly: In a study that examined the locus of deficit for 40 randomly selected adults with dysnumeria, all except one were impaired in a syntactic process, whereas only 14 of them (35%) were impaired in a lexical process (some participants had both impairments; Dotan & Handelsman, in prep.).

Understanding the cognitive underpinnings of syntax, not only that of numbers but also in general, is important not only for its real-world impact but also as a central theoretical question in cognitive psychology. Representing complex syntactic information, which encodes not only the identity of each item but also the relations among items, seems to be a considerable cognitive challenge in several different domains. Cognitive representations of syntactic relations exist in numbers; in language, to represent the grammatical inter-dependencies of the words in a sentence, (Chomsky, 1956); in arithmetic, to represent the hierarchical structure of algebraic expressions (Schneider et al., 2012; van de Cavey & Hartsuiker, 2016; Zeng et al., 2018); to represent the relational rules underlying arrays of shapes (Pothos & Bailey, 2000), sounds (Gentner et al., 2006; Horváth et al., 2001), spatial positions (Al Roumi et al., 2020), or other stimuli; and even to represent and plan motor action (Koechlin & Jubault, 2006; Moro, 2014). Some forms of syntax are simpler than others, but some syntactic representations—in particular, those organized as a hierarchy of elements—seem to be quite complex, and to a large extent—human-specific. Indeed, some animal species, e.g., songbirds (Berwick et al., 2011; Gentner et al., 2006), may be able to handle even relatively complicated syntactic structures, including some hierarchical structures, but only humans can handle complex hierarchical structures in a flexible manner and combine them with their meaning, as we do in the case of language or numbers (Dehaene et al., 2015; Hauser et al., 2002). Understanding how people process the syntactic structure of numbers may potentially illuminate on how humans process syntactic information in general.

### What we already know about the processing of number syntax

“Number syntax” is not a unitary cognitive construct, handled by a single process—there are several different processes that handle different aspects of number syntax. We already know quite a bit about the low-level processes that handle highly specific syntactic aspects of numbers. These processes can be roughly classified according to the type of information being handled (digits versus number words) and the processing stage (input/comprehension versus production). In the digit-input mechanisms, i.e., when parsing a visually presented digit string, there are separate processes to handle the string length (how many digits it has), the positions of 0, the grouping of digits into triplets, and the relative order of digits (Cohen & Dehaene, 1991; Dotan & Dehaene, 2020; Dotan & Friedmann, 2018; Dotan et al., 2021b). In digit production mechanisms, i.e., when writing digit strings, dedicated processes handle the positioning of 0 (Furumoto, 2006) and the order of digits (Lochy et al., 2004). In oral production of verbal numbers, specific processes handle the number words’ lexical classes (ones, tens, teens, etc.), which are essentially the syntactic aspect of the verbal number (Cohen & Dehaene, 1991; Dotan & Friedmann, 2018, 2019; McCloskey et al., 1986); other processes bind each digit with the appropriate lexical class (Blanken et al., 1997; Dotan & Friedmann, 2018); and yet other processes retrieve the morphological affix corresponding with each lexical class (Cohen et al., 1997; Dotan & Friedmann, 2015). Finally, when comprehending a verbal number, specific syntactic processes handle the place-value information (Kallai & Tzelgov, 2012; Lambert & Moeller, 2019), the order of words (Hayek et al., 2020; Zuber et al., 2009), and the merging of adjacent pairs of number words into a single syntactic structure when this is grammatically possible (as in *thirty-two*, but not in *two-thirty*, Hung et al., 2015).

On top of these low-level syntactic processes, there exists a core representation of the number’s full syntactic structure. Namely, the number’s full syntactic structure is represented explicitly in the brain, and the human ability to handle number syntax is not just a by-product of other types of representations, e.g., some lower-level syntax-related processes. This representation, on which the present study focuses, was a central idea in the number-processing model of McCloskey and his colleagues (McCloskey, 1992; McCloskey et al., 1986). Specifically, they proposed that multi-digit numbers have a central abstract representation, which incorporates the full information about the number’s semantics and syntax. McCloskey’s model made an extreme assumption—that this representation incorporates both the number’s syntax and its semantics, and that it mediates any task involving any symbolic numbers (digits or words), including reading, writing, comprehension, production, and calculation. This extreme assumption was refuted (Campbell & Clark, 1992; Cohen & Dehaene, 1991, 2000; González & Kolers, 1982; Noël & Seron, 1997). The refutation has led several researchers to abandon McCloskey’s model in favor of other cognitive models of number processing—especially Dehaene’s triple-code model (Dehaene, 1992; Dehaene & Cohen, 1995; Dehaene et al., 2003), which focuses on the different representations of numbers and remains largely silent about the issue of number syntax and about the differences between single-digit and multi-digit numbers. However, a recent study (Dotan et al., 2021a) supports a weaker version of McCloskey’s assumption. In this study the participants heard, on each trial, a number between 1 and 9999 and responded by saying a random number in the same range. The syntactic structure of their responses was similar to that of the target numbers—a syntactic priming effect, which indicates that they represented the number’s syntactic structure. The researchers concluded that a representation of the number’s full syntactic structure exists—perhaps not for any number and in any task, but at least in some tasks and at least for numbers up to 4 digits long.

Another interesting idea in McCloskey’s (1992) number-processing model is that the syntactic representation of numbers has a hierarchical, tree-like structure: The units and decades are merged first; then, this pair is merged with the hundreds (thereby forming a triplet), and finally two triplets can be merged. For example, the number 234,567 would be represented as *[2 & (3 & 4)] & [5 & (6 & 7)]*. Such hierarchy resembles the way we represent sentences (Chomsky, 1956, 1995) and other types of information (Dehaene et al., 2015). At present, this hierarchical representation is still an unconfirmed hypothesis. As we shall see, the present study will bring several pieces of suggestive evidence in favor of this idea.

### What we don’t yet know about the processing of number syntax

The aforementioned studies provide a relatively good picture of many peripheral syntactic processes—in particular, those involved in parsing the syntactic structure of sequences of digits or number words, and in the production of digit strings and multi-digit verbal numbers. In contrast, little is known about the core representation of number syntax. The present study aims to fill this gap: Our general goal was to identify several characteristics of a representation of the full syntactic structure of numbers and of the processes that create it.

Specifically, our first goal was to reaffirm the existence of a core representation of the syntactic structure of numbers. To our best knowledge, to date only a single study showed that such a representation actually exists (Dotan et al., 2021a). Here, we will start by replicating this conclusion using another paradigm.

A second question concerns the flexibility of the syntactic representation. An influential idea in syntactic theory is that certain types of complex syntactic structures, which are unique to humans, are not predefined rigid cognitive structures; rather, they are created in a generative manner by operating recursively on the syntactic representation (Hauser et al., 2002). Here, we examined whether the syntactic representation of numbers is created dynamically by a generative process, or is a rigid predefined representation. According to the former view, whenever we process a number, we recreate its syntactic structure in a generative step-by-step manner. This view is in excellent agreement with the notion that the syntactic structure of numbers is represented in a hierarchical tree-like manner (McCloskey, 1992; McCloskey et al., 1986). According to the second view, the number’s syntactic structure is a predefined memorized “template,” in which we embed the digits, and this representation is retrieved from a mental lexicon of number-syntax templates. The “lexicon of templates” view is not unlikely, especially given the small number of syntactic structures: For example, based on the common definition of syntactic structure as a series of number-word lexical classes (ones, tens, teens, etc.), English numbers with 1–3 digits have only 9 different syntactic structures: ones (e.g., for 5), tens (50), teens (15), tens ones (55), ones hundred (500), ones hundred ones (505), ones hundred tens (550), ones hundred teens (515), and ones hundred tens ones (555).

A third question pertains to the scope of the syntactic representation. In the single study that showed a core syntactic representation (Dotan et al., 2021a), the stimuli were Hebrew and Arabic verbal numbers up to 9999. Such numbers are limited in two ways. First, their syntactic structure is relatively simple. In spoken Hebrew and Arabic, numbers up to 9999 do not make use of the multiplier words “hundred” and “thousand” as English numbers do. Rather, ones, tens, hundreds, and thousands are four different lexical classes (e.g., in Hebrew, 3 = /shalosh/, three; 30 = /shloshim/, thirty; 300 = /shloshmeot/; 3000 = /shloshtalafim/, and similar in Arabic; see Supplementary Material for additional details about the Hebrew verbal number system). Thus, in a number up to 9999, the different words always belong to different lexical classes—the same class never appears twice. Only numbers with 5 digits or more have the English-like hierarchical structure, in which the word “thousand” separates two similarly structured phrases (e.g., “twenty-three thousand forty-five”). It thus remains to be shown whether the numbers’ core syntactic representation can handle the hierarchy-like aspect induced by the multiplier words “hundred” and “thousand,” or is limited to the simpler forms of syntax.

The second limitation of Hebrew and Arabic numbers up to 9999 is that they have up to 4 words, so they can potentially fit in a single chunk in working memory (Cowan, 2001, 2010). Can the syntactic representation exceed the size of a single chunk in working memory? Arguably, the ability to transcend a single chunk is one important advantage of hierarchical representations.

A fourth and final question is whether number syntax is created automatically and without directed attention, similar to syntactic structures in several other domains, e.g., language and music (Batterink & Neville, 2013; Maidhof & Koelsch, 2011), or must it be created voluntarily, via a process that requires our intention and attention.

The four issues above were presented here as theory-driven questions, but they also have concrete pedagogical implications. For example, if syntactic structures are rigid templates (question 2), the best way to teach children the syntax of numbers may be by memorizing the list of templates, whereas if syntax is generative, a better method may be to teach the generative syntactic rules. If syntax is created via attention-requiring processes (question 4), it may be best to teach overt strategies to represent syntax, but if it is created by automatic processes, training and rehearsal might be the better pedagogical approach. We revisit these pedagogical implications in the General Discussion.

### The present study

We used a paradigm we called Syntactic Chunking. In each trial, the participants heard a sequence of number words and repeated it. The number of words in each stimulus (sequence) was constant, but critically, we systematically varied the stimulus grammaticality: In some conditions, the stimulus consisted of a single grammatical segment (e.g., *two hundred thirty four*), and in other conditions the stimulus included several, shorter grammatical segments (*thirty four two hundred*), sometimes even fragmented almost entirely to single-word segments (*hundred two four thirty*). If the participants represent the syntactic structure of each grammatical segment, repetition accuracy should be better in the conditions with longer grammatical segments than in the more-fragmented conditions, because a syntactic representation may help merge the words of each segment into a single chunk in short-term memory, and this chunking should improve the participant’s memorization (Cowan, 2001; Miller, 1956). Critically, chunking in working memory is typically not arbitrary but depends on the specific stimulus at least in two ways: First, the specific stimulus may affect the selection of the chunk boundaries. Second, the stimulus determines the degree of compressibility, with more compressible stimuli enabling the creation of chunks that contain more data, thereby improving memorization (Mathy & Feldman, 2012). In our case, we assumed that both chunk boundaries and compressibility would be driven by the number’s syntactic structure, which allows creating strong associations between the words in a grammatical segment. Such associations facilitate chunking (Cowan, 2001).

A similar manipulation was used in two previous studies (Barrouillet et al., 2010; Hung et al., 2015). Similar to us, both studies manipulated the degree of grammaticality in number-word sequences; however, they also differed from the present study in critical respects. Barrouillet et al. used children, whereas we focused on the automatic processing of numbers in literate adults. Hung et al. used adult participants, but there were critical differences between their methodology and analyses and ours, and consequently, their study and ours tap different stages of syntactic processing. We return to these issues in the General Discussion, where we explain in detail the similarities and differences between these studies and ours, and how the 3 studies complement each other.

## General methods

### Participants

The participants in all experiments were adults without any reported cognitive deficits. They were native speakers of Hebrew, and the experiments were run in this language. They were compensated for participation.

### Screening

As screening, we examined each participant’s short-term memory using a digit span task (Friedmann & Gvion, 2002)—repeating digit sequences in increasing length. There were 5 sequences for each length from 2 to 9 digits. The participants proceeded to the next length if they repeated accurately 3 out of the 5 sequences. The span is defined as the longest sequence length in which the participant repeated 3 sequences correctly, with additional half a point if they repeated 2 sequences of the last length. The average span of adults (age 20–30) in this task is 7.05 (SD = 0.94). We included only participants with span 6 or higher.

### Syntactic chunking task

In each trial, the participant heard a sequence of number words, said a short, fixed sentence in Hebrew (“what a nice day it is”), and then repeated the number words. Saying the sentence was aimed to “reset” the phonological short-term memory and to reduce the likelihood of phonological repetition strategies in favor of strategies based on a whole-number representation. The participants were encouraged to provide partial information about the stimulus if they did not remember it fully. Each stimulus (sequence of words) was presented only once. In case of an interruption, the trial was canceled and presented again at the end of the block.

The critical manipulation was the stimulus grammaticality. In a fully grammatical condition, each stimulus—a sequence of number words—formed a single grammatical segment (e.g., *two hundred fifty seven*). In the more-fragmented conditions, each stimulus consisted of several grammatical segments. For example, the stimulus *fifty seven two hundred* forms two grammatical segments, *fifty seven* and *two hundred*. Below, we use the term *segment* to denote a grammatically valid subsequence of the stimulus, which is also maximally valid—i.e., the segment ends when grammaticality ends. For example, the sequence *fifty seven* cannot be considered as two separate single-word segments, because these two words, in the given order, can be merged grammatically.

## Experiment 1

### Method

The participants were 20 adults aged 20;2–36;0 (mean = 25;6, SD = 3;9).

#### Syntactic chunking task

**Fig. 1:**
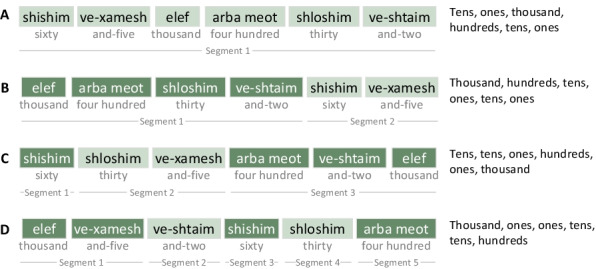
Experiment 1 design. The participants heard sequences of number words in Hebrew, and repeated each sequence. Each of the 4 conditions included 20 stimuli (sequences), derived from the same 20 numbers (in this example: 65,432). The stimulus words were shuffled in a different manner in each condition, according to that condition’s fixed word order (shown here on the right). Thus, the number and length of grammatical segments was fixed for each condition, and different between conditions. A stimulus included either 6 words (as in this example) or 7 words, depending on the participant’s short-term memory capacity. For the 7-word stimuli, the word order in each condition was **A** [hundreds, tens, ones, thousand, hundreds, tens, ones]; **B** [thousand, hundreds, tens, ones], [hundreds, tens, ones]; **C** [hundreds, tens], [hundreds, ones], [tens, ones, thousand]; **D** [thousand, ones], [ones], [tens], [tens], [hundreds], [hundreds]

The experiment had 4 conditions, administered in 4 blocks. In condition A, each stimulus was a single grammatical segment, which included only the digits 2–9 and did not include the same digit twice. In conditions B, C, and D, each stimulus consisted of more, shorter grammatical segments (Fig. 1). All stimuli in a given condition had the same syntactic structure. To control for lexical effects, all 4 conditions included the same 20 sets of words; they differed only in the order of words within each stimulus.

The participants’ ability to remember the stimuli is presumably affected not only by the syntactic properties of the stimulus, but also by their individual short-term memory capacity. Thus, the number of words in each stimulus was determined according to the participant’s digit span: Participant with span 6 heard 6-word stimuli (corresponding with 5-digit numbers), and those with span 7 heard 7-word stimuli (corresponding with 6-digit numbers).

The syntactic structure of number words in Hebrew is similar to that of English. The only difference relevant for this experiment is that whereas in English, the phonological form of each hundreds word consists of two separate words (e.g., “three hundred”), in Hebrew each hundreds word is presumably a single lexical entry (e.g., 300 = /shloshmeot/, “threehundred”). As a result, it is easier to create fully fragmented sequences of words in Hebrew than in English—we merely sorted the words according to their lexical classes—first the Ones words, then the Tens words, then the Hundreds words. For example, the number 234,567 would appear in the most-fragmented condition as *thousand, four, seven, thirty, sixty, twohundred, fivehundred*. To prevent any experimenter-originated bias (e.g., difference between the conditions in intonation), each number word was recorded separately, and single-word recordings were merged with a 200 ms gap between words into a full auditory stimulus.

The participants of Experiment 1 also performed Experiment 2 (described below). Each participant was randomly assigned to one of two orders of the blocks, and to a random order of Experiment 1 versus Experiment 2. The specific orders were: ABCD2, DCBA2, 2ABCD, or 2DCBA. In Experiment 1, each block started with short training: The experimenter said explicitly the word-order of that block, and then the participant performed 2 training trials with that block’s syntactic structure.

#### Data coding

Each number word is uniquely defined by a lexical class (in this experiment we used only ones, tens, or hundreds) and a 1–9 value. For example, the word *fifty* is the combination of the digit 5 and the class Tens. Similarly, “four hundred” (presumably a single lexical-phonological value in Hebrew) is the digit 4 in class Hundreds. Correspondingly, the cognitive representation of number words is in two morphemes, digit and class (McCloskey et al., 1986), so our coding was based on these two morphemes. The decimal word “thousand” was an exception: We considered it as a single morpheme, the lexical class “thousand” with no digit morpheme.

We defined 3 performance measures for each trial, reflecting accuracy in the digit morphemes, in the class morphemes, or in both. *Digit accuracy rate* was defined as the percentage of stimulus digits that appeared in the response, irrespectively of their order and ignoring excessive digits. The word “thousand” was excluded from this measure. *Class accuracy rate* was defined as the percentage of stimulus lexical classes that appeared in the response, irrespectively of their order and ignoring excessive classes. If the stimulus included a lexical class twice (e.g., the tens class in “ninety thousand and eighty”) but the response included it only once, it scored only 1 accuracy point out of 2. Finally, *morpheme accuracy rate* was defined by merging the two above—i.e., the percentage of digit and class morphemes that appeared in the participant’s response, irrespectively of their order. In the text below, we report the morpheme, digit, and class *error* rates, i.e., the complement to 100% of the accuracy rates.

A fourth possible measure is the *word accuracy rate*—the percentage of stimulus words that appeared in the response. Similar to morpheme accuracy rate, this measure too considers both the class and the digit, but it also requires correct pairing of a particular class with a particular digit. For example, repeating *twenty-three* as *thirty-two* would be coded as 0% word accuracy and as 100% morpheme accuracy. The word accuracy results are not reported here, but they were essentially the same as the morpheme accuracy results.

#### Statistical analysis

To compare between two conditions, we entered the digit, class or morpheme error rate of each trial as the dependent variable in a linear mixed model (LMM). Participant and Stimulus were random factors, and the condition was a within-participant, within-stimulus factor. In the few cases in which a model did not converge, we removed the Stimulus random factor. To control for the fact that different participants repeated stimuli of different lengths, Stimulus Length (the number of words) was entered as a covariate. We used R (R Core Team, 2019) with the *lme4* package (Bates et al., 2015). To determine whether the effect of Condition was significant, we used a likelihood ratio test that compared the LMM to an LMM that was identical except it did not include the Condition factor. For these comparisons, we report the test statistic 2(LL_1_–LL_0_), which follows a *χ*^2^ distribution (LL_0_ and LL_1_ denote the log-likelihoods of the reduced model and the full model), and the corresponding *p* value. The degrees of freedom are not reported as they were always 1. To compare the performance of a single participant between two conditions, we used the same method, but the model did not include the Participant and Stimulus Length factors; it included only Stimulus as a random factor and Condition as a within-stimulus factor. As effect size, we report the Condition factor’s coefficient in the model. This coefficient is close to the difference between the conditions’ means, so it is denoted Δ.

### Results

Four participants performed the 7-word version of the task, and the remaining participants performed the 6-word version.

**Fig. 2:**
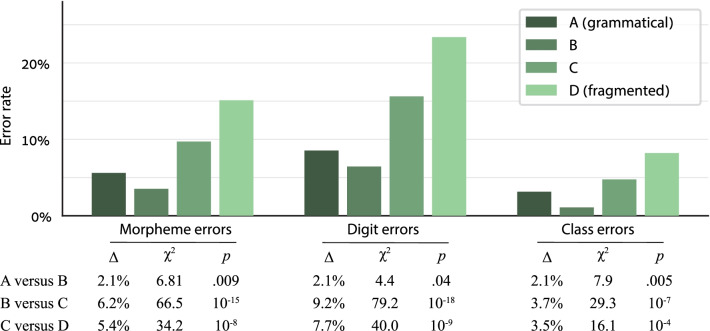
Experiment 1 results: the morpheme, digit, and class error rate in each condition. The best performance was in condition B. In this condition, each stimulus included 2 grammatical segments with 3–4 words in each; this seems to provide the optimal balance between the number of chunks and the chunk size. The performance was poorer in conditions C and D, which seem to support too little chunking as the stimuli have short grammatical segments; and in condition A, which seems to encourage too much chunking as the stimulus has a single long grammatical segment. This clear syntactic chunking effect shows that the participants represented the number’s syntactic structure. In the statistics table, Δ, *χ*^2^, and *p* refer to the effect size and significance of the Condition factor in the linear mixed model described in the “Statistical analysis” section

The simplistic prediction of syntactic chunking is that if a stimulus has more or longer grammatical segments, it should be easier to remember. However, longer segments may be disadvantageous if they are too long; such segments may result in creating mega-chunks that exceed the working memory capacity limit and are hard to remember. Thus, the relation between segment size and performance is expected to have an inverted U-shape: The best performance should not be in the stimuli with the longest segments, but in the stimuli whose segment length offers an optimal balance between the number of chunks and the chunk size. Conditions with too-many, too-short grammatical segments would induce relatively little chunking and lead to ineffective memory strategies; and conditions with too-few, too-long segments would encourage the creation of oversized, hard-to-remember chunks. As Fig. 2 clearly shows, this was precisely the case: Accuracy was highest in condition B and lowest in the other conditions. In particular, the error rate in condition B was significantly higher than in condition C, which in turn had significantly more errors than in condition D—a clear effect of syntactic chunking. Namely, although the participants received no particular instructions about chunking strategies, they still used the number’s syntactic structure and created chunks that represent grammatical multi-digit numbers. As expected, the optimal performance was not in condition A, in which the grammatical segments were the longest, but in condition B, which seems to offer the optimal balance between chunk size and number of chunks.

**Fig. 3:**
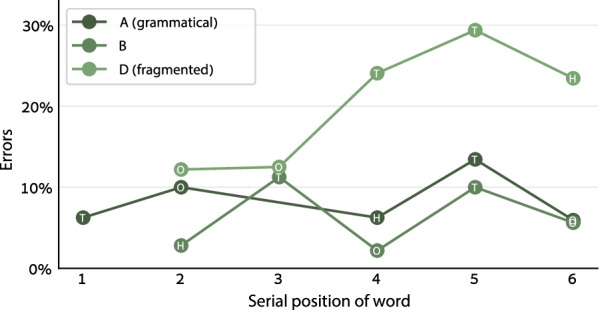
Digit error rate in the 6-word numbers in Experiment 1 for each serial position of the number words. In condition D, the error rate was the lowest for the first two words and increased thereafter (except the last word)—a standard pattern of primacy effects in free recall tasks. Conditions A and B did not show this pattern: In these conditions, the error rate did not show a consistent pattern. The word “thousand” is not plotted here, as its digit error rate is undefined. The letters H, T, and O denote a Hundreds, Tens, and Ones word, respectively

Additional support for the idea of syntax-based chunking comes from analyzing the specific positions within the stimuli in which the errors occurred. Figure 3 shows the digit error rate in each serial position for the participants who repeated 6-word sequences (the 7-word participants were excluded from this analysis to avoid length-related variance). If the participants memorized each stimulus as an unstructured sequence of words, the task is essentially a free recall task. In such task, the error rate should typically be the lowest for the initial words in the list and gradually increase for words further down the list (a primacy effect), with some improvement in the last word or words (a recency effect; Murdock, 1962). Condition D, the fragmented condition, shows this pattern of unstructured free recall tasks. In contrast, conditions A and B show a different pattern, indicating that additional factors were at play here on top of the primacy and recency effects. For example, in condition B the error rate *decreased* from the 2nd word (thousands) to the 3rd word (hundreds). To examine the pattern in each condition, we analyzed the digit accuracy in each number word in the 6-word numbers, excluding the word “thousand” (for which no digit was encoded) and excluding the last non-thousand word in each number (to avoid the recency effect). We submitted the digit accuracy of each condition separately to a logistic linear mixed model with Participant as a random factor and the word’s serial position as a numeric within-participant factor. We did not add the Stimulus as a random factor because such model reached a singular fit in some conditions, but the results were essentially the same when including this factor. The Word Position effect was significant in condition D (*χ*^2^ = 27.4, *p* < 0.001) but only barely significant in conditions A (*χ*^2^ = 4.7, *p* = 0.03) and B (*χ*^2^ = 4.2, *p* = 0.04)—unimpressive significance levels that do not withstand a multiple-comparison correction. To show that the difference between conditions was significant, we submitted the data of all conditions together to the same LLMM, now adding the Condition (A/B versus D) and the Condition × Word Position interaction as within-participant factors. The interaction term was significant (*χ*^2^ = 11.5, *p* < 0.001, odds ratio = 0.75).

Simple serial recall cannot explain the results in conditions A and B, however, syntactic chunking offers a simple explanation to this pattern: In condition D, the participants remembered each stimulus as an unstructured list of words, but in conditions A and B they tended to memorize each stimulus as chunks. Because of this chunking the task was not really a simple serial recall task, so it did not show a standard primacy and recency effects. Interestingly, in conditions A and B a primacy effect was observed not only for the sequence as a whole: In both conditions, the performance in word #4 was better than in the preceding and in the next word, and words 4–5–6 showed an inverse U-shaped pattern. This pattern is in line with the idea that words 4–5–6 were encoded as a separate chunk, with its own primacy and recency effects in the chunk’s first and last words.

**Fig. 4:**
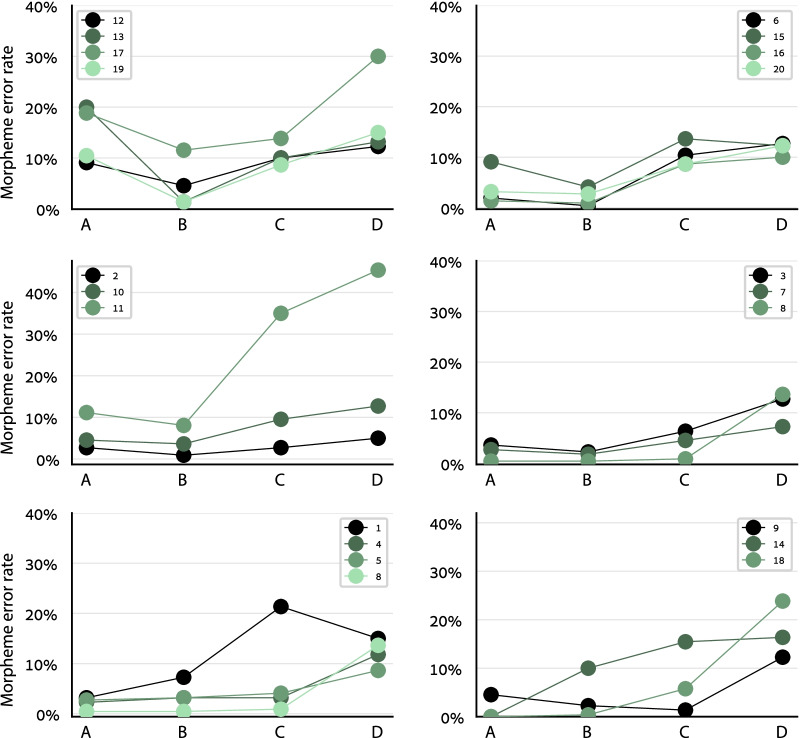
Individual participant results in Experiment 1. Each line shows one participant (participant ID in the legend). The syntactic chunking effect, in particular the difference between conditions B and D, is clearly visible for each individual participant and was significant for most of them. The best-performance point was condition B for most participants, but for some it was condition A or C (bottom panels)

The syntactic chunking pattern—better performance in the more-fragmented conditions, and optimal performance in an interim condition—was observed not only at the group level but even for individual participants. Numerically, each of the participants showed better performance in condition B (optimal chunking) than in condition D (maximum fragmentation) (Fig. 4). This difference was significant for all participants except one (morpheme accuracy rate of each of these participants: paired *t*(19) > 1.73, Bonferroni–Holm corrected one-tailed *p* < 0.05). Because the different conditions used the same stimuli and manipulated only the word-order within each stimulus, we could also compare matched pairs of stimuli and show that a syntactic chunking effect existed even for single stimuli in most cases: Morpheme accuracy was better in condition D than in B only for 6% of the stimuli (and better in B for 63.5%; same in B and D for 30.5%). Nevertheless, the participants also differed from each other—the best-performance condition was different for different participants: For 13 participants, the optimal condition was B, but for 6 participants it was A and for one participant it was C (bottom panels in Fig. 4).

### Discussion

The best performance was in condition B, in which each stimulus was a pair of 3-word or 4-word segments. In conditions C and D, the performance deteriorated as the stimulus included more, shorter grammatical segments. This syntactic chunking effect indicates that the participants created a representation of the syntactic structure of whole numbers, and this representation allowed them to create increasingly longer chunks for increasingly longer grammatical segments.

The best performance was not in condition A, which had a single long segment per stimulus, but in condition B. Namely, although the performance improved from condition D to C and from C to B, longer segments (in condition A) disrupted memorization. This pattern of results supports the notion that effective chunking requires optimal balance between the chunk size and the number of chunks. It seems that in condition A, the participants used the full syntactic structure of the 5- or 6-digit number to store all words in a single chunk, and this led to exaggerated chunk sizes, and consequently to poorer memorization. According to this view, although the error rates had a U-shaped pattern, the underlying syntactic chunking was actually a monotonous effect: Longer grammatical segments *always* led to larger chunks, including in condition A, but increasing the chunk size was beneficial only up to an optimal threshold size of 3–4 words per chunk, which occurred in condition B. Beyond that, in condition A, the increased chunking disrupted performance because the chunks became too large for the participants to handle effectively—they exceeded the working memory limit that each chunk is subject to Cowan (2001). Below, in Experiment 5, we bring additional evidence to support this conclusion.

Our findings refute an alternative interpretation that attributes the difference between condition A and condition B to number magnitude. This alternative interpretation postulates that the performance in condition A was poorer than in B not for reasons related to syntax and chunking, but because the numbers in condition A were numerically larger and therefore harder to process (a size effect). Two aspects of our data refute the alternative interpretation: First, it cannot explain the error-by-position pattern in Fig. 3. Second, the alternative interpretation predicts that 5- or 6- digit numbers (as in condition A) will *always* be more difficult to memorize than a pair of shorter numbers (condition B), irrespectively of the specific numbers. As we shall see in Experiment 5, this prediction was refuted.

The superior performance in condition B relative to condition A leads to several important conclusions. First, it shows the scope of the syntactic representation of numbers—in particular, a representation of cross-triplet syntax. In conditions B, C, and D, no grammatical segment crossed the bounds of a single triplet (hundreds + tens + ones). The differences between these conditions can be explained as a within-triplet syntactic representation—for example, a representation capitalizing on the fact that the three words in each triplet have different lexical classes. The situation was different in condition A versus B: The only difference between these two conditions was that condition A, but not B, combined words from the two triplets into a single segment. The significant difference between the two conditions indicates that in condition A, but not in B, the participants created a cross-triplet syntactic representation. In the General Discussion, we return to the implications of this finding.

The second conclusion concerns automaticity. In condition A, the cross-triplet syntactic representation was created even though it was not beneficial—it actually disrupted the performance. This strongly suggests that the creation of a syntactic representation did not result from a voluntary, conscious-strategic decision, but was an automatic process.

The third conclusion pertains to whether the syntactic representation is retrieved as a rigid template or created dynamically by a generative process. Cowan (2001) proposed that there are at least two different methods to create chunks, and these two methods differ in the limit they impose on the chunk size. One method is to retrieve a memorized template, which serves as the basis for the chunk, and embed several single items in this template (each single item is a representation from long-term memory). For example, this may be how expert chess players encode complex moves. The number of items in such templates may sometimes be quite large—more than the standard short-term memory capacity of 3–4 items—but the template is still a single chunk. A second method to create chunks is by forming novel ad hoc associations between items. This method is more dynamic and flexible, but the cost is that the chunk size is subject to the working memory capacity limit of 3–4 items. The critical point is that these two methods differ in the restrictions they impose on the chunk size: The latter method is subject to working memory capacity limits, whereas the former is not. Thus, in our experiment, if the syntactic chunks are created in a generative manner, they should be subject to the working memory capacity limit, leading to poor performance in oversized chunks—precisely the pattern we see in condition A. Had the number’s syntactic structure been a memorized template, the participants could have created template-based chunks, which are not subject to the capacity limit, and the performance in condition A (with 1 chunk per stimulus) should have been better than in condition B (2 chunks per stimulus). Clearly, this was not the case. We therefore conclude that the number’s syntactic structure was not retrieved as a predefined memorized template, but was created by a generative process in real time.

## Syntactic chunking genuinely indicates a syntactic representation

Efficient chunking involves two key aspects of the stimulus: detectability and compressibility (Chekaf et al., 2016). The first aspect pertains to the detection of regularities in the stimulus, which provide opportunities for effective chunking, e.g., by setting optimal chunk boundaries. In our case, this would refer to the detection of the grammatical segments. The second aspect refers to the process that actually compresses the data into a chunk, presumably by relying on some representation with strong associations between the chunk’s elements (Cowan, 2001). In our case, compressibility is presumably driven by the representation of the number’s syntax.

We interpreted Experiment 1 results in terms of compressibility: We argued that the critical difference between the experimental conditions was that they affected the participants’ ability to create syntax-based chunks. Could it be, however, that the conditions actually differed from each other in the *detectability* of grammatical segments? In Experiment 1, we intentionally did not provide any cues for chunking (so not to give any clue that may affect detectability), but in the absence of such cues, the participants may have resorted to other strategies that could give rise to different detectability levels in different conditions. For example, they may have used a simple strategy such as “closing a chunk” after each Ones word—a strategy that may result in more efficient chunking in condition B than in condition D. Alternatively, they may have used overt strategies based on their formal mathematical knowledge, and such strategies may be easier to implement in the grammatical conditions, which presumably have better fit to the participant’s formal knowledge about numbers. The effects of detectability may have been further amplified by the blocked design of Experiment 1, which may allow developing condition-specific strategies in each block.

The comparison between conditions A and B in Experiment 1 suggests that this was not the case, because the participants created a syntactic representation even when this did not pay off (in condition A). Nevertheless, we designed Experiments 2 and 3 to specifically refute the alternative interpretation. In Experiment 2, we used a mixed design to discourage block-specific strategies. In Experiment 3, we provided clear cues about the chunk boundaries, in order to minimize the detectability differences between the conditions.

### Experiment 2: mixed design

The design was similar to Experiment 1, but here the different conditions were mixed in a single block. This design should make it hard to use overt strategies, because the participants could not know the syntactic structure of the specific stimulus until after it was played out. Thus, a syntactic chunking effect in Experiment 2 would be hard to explain as resulting from overt strategies.

#### Method

There were 3 conditions with 2, 3, or 4 grammatical segments per stimulus, with 4, 9, and 7 trials in each condition, respectively. All trial types were presented in a single block, in random order (same order for all participants). The participants were the same ones who performed Experiment 1; they performed Experiment 2 immediately before or after Experiment 1 (see “Syntactic chunking task” section). Each participant performed either the 6-word or the 7-word version of the task, as in Experiment 1. The data coding and the statistical analysis were as in Experiment 1, but in the linear mixed model, the Condition factor was a between-stimulus numeric factor rather than a within-stimulus categorical factor. (It was still within-participant.)

#### Results and discussion

**Fig. 5:**
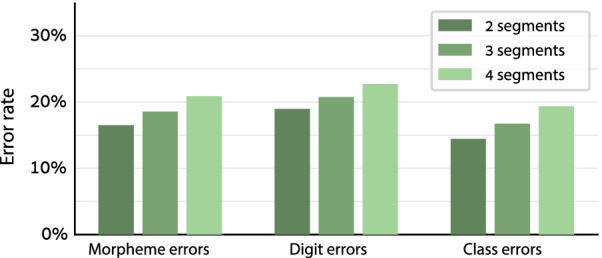
Experiment 2 results: the morpheme, digit, and class error rate in each condition. Error rates were lower in the conditions with fewer, longer segments, which allowed for more syntax-based chunking

The results of Experiment 1 were essentially replicated: The performance was better in the conditions with fewer, longer segments (Fig. 5). The linear mixed model showed that the Condition effect was significant for the morpheme error rate (Δ = 2.2%, *χ*^2^ = 4.17, *p* = 0.04) and the class error rate (Δ = 2.5%, *χ*^2^ = 5.09, *p* = 0.02), although not for the digit error rate (Δ = 1.9%, *χ*^2^ = 2.48, *p* = 0.12).

These results are hard to explain as an overt strategy. For such overt strategy to be effective, the participants would have had to determine their strategy on each trial, and to do so only after the stimulus was played out, at which time the memorization challenge had already began. The better and more likely explanation is that grammatical conditions were advantageous due to syntactic chunking.

### Experiment 3: overt cueing

This experiment specifically aimed to rule out grammatical-segment-detectability as an explanation of the syntactic chunking effect. To this end, we turned detectability into a non-issue by making it as easy as possible in all conditions: We gave the participants clear, explicit cues about how they should split the sequence of words into chunks. If the difference between the conditions in Experiment 1 originated in the detectability of grammatical segments, the overt cues should override any subtle stimulus-specific differences, and the performance should be similar across conditions. If, however, Experiment 1 results resulted from the higher compressibility of grammatical segments, the results should be replicated here too.

#### Method

The participants were 20 adults aged 19;6–52;7 (mean = 27;7, SD = 7;9). The experiment included only two conditions, identical (same stimuli) with Experiment 1 conditions B (grammatical) and D (fragmented). All participant performed the 7-word version of the task. The conditions were administered as two blocks in counterbalanced order. The procedure was as in Experiment 1, except that now we provided clear cues for the chunk boundaries. Critically, the cues were identical in both conditions (grammatical and fragmented): In both cases, the participants were cued to split each stimulus into two chunks, one with 3 words and one with 4 words. In the grammatical condition, the cue split the stimulus into a 4-digit number starting with the digit 1, followed by a 3-digit number (e.g. *thousand twohundred thirty four; fivehundred sixty five*). In the fragmented condition, the cue split the stimulus into two parts of the same length, but neither part was grammatical: The order of words was reversed to avoid any grammatically valid pair of words (*five sixty fivehundred; four thirty twohundred thousand*).

As cueing, we used two aspects of intonation. First, the experimenter did not say the number words in fixed pace as in Experiment 1, but as we would say two numbers: There was no delay between the words within each stimulus part, and a delay of about 1 s between the two parts of the stimulus. Second, the last word of each stimulus part was said with descending pitch (intonation typical to the last words of sentences), and all preceding words were said in flat intonation. To facilitate attention to these cues, the stimuli were not played from recording as in Experiment 1, but were said in real time by the experimenter. The data coding and the statistical analysis were as in Experiment 1.

#### Results and discussion

**Fig. 6:**
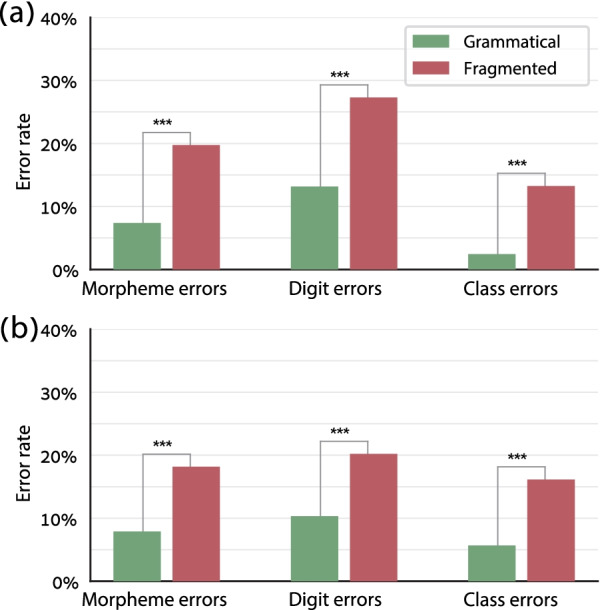
Results of Experiments 3 (**a**) and 4 (**b**). The morpheme error rate, digit error rate, and class error rate were lower in the grammatical conditions than in the fragmented ones. Asterisks denote the significance of the Condition factor in the linear mixed model described in the text (all *p* < 0.001)

The results of Experiment 1 were essentially replicated: The morpheme error rate, digit error rate, and class error rate were lower in the grammatical condition than in the fragmented condition (Fig. 6a; with the LMM described in Experiment 1 “Statistical analysis” section, morphemes: Δ = 12.3%, *χ*^2^ = 140.5, *p* < 0.001; digits: Δ = 12.1%, *χ*^2^ = 136.1, *p* < 0.001; classes: Δ = 10.8%, *χ*^2^ = 103.8, *p* < 0.001). Even at the single-subject level, the morpheme error rate was lower in the grammatical condition than in the fragmented condition for each single participant (Additional file 1: Fig. S2).

The difference between the two conditions, which existed although we provided very clear cues for how to divide each stimulus into two chunks, is unlikely to have arisen from different degrees of grammatical-segment-detectability in the two conditions. The most likely interpretation of these results is that the grammatical condition, by using valid number syntax, allowed for higher compressibility of the number-word sequence.

## Experiment 4: the syntactic representation can handle varying irregular structures

Experiments 1–3 showed that the participants represented the number’s syntactic structure and used it as the basis for chunking. Experiment 4 examined two additional aspects of this syntactic representation: its scope, i.e., the specific syntactic structures that can be represented, and its flexibility, i.e., the ability to quickly switch from one syntactic structure to another. To this end, we included numbers with several different syntactic structures.

**Scope. **Experiments 1–3 used a limited scope of numbers: All stimuli were based on numbers that included neither 0 nor 1. This design aimed to avoid a complexity that may arise from numbers with 0 and 1, because these two digits create verbal numbers with irregular syntactic structures: In Hebrew, similar to English, the digit 0 is not realized verbally, and the digit 1 in the decade position is realized verbally as a teens word instead of the standard tens word. By avoiding 0 and 1, Experiments 1–3 included only numbers with regular syntactic structures, and no numbers with irregular structures. In contrast, Experiment 4 examined whether a syntactic representation would be created also for irregular numbers.

**Flexibility. **The second aspect we examined is the flexibility of the syntactic mechanisms. In Experiments 1 and 3, all numbers in a given block had the same syntactic structure. Experiment 2 had a mixed design, but it still used only a small variety of syntactic structures. Experiment 4 used more syntactic structures and presented them in random order, so we could examine whether the syntactic representation, and the processes that create it, are flexible enough to serve as the basis for syntactic chunking even in this more-demanding scenario.

### Method

The participants were the same 20 adults who performed Experiment 3. The design was similar to Experiment 3: There were two conditions, grammatical and fragmented, and we used overt cueing. Only the specific stimuli differed from Experiment 3. The stimulus of each trial included 5, 6, or 7 words, and critically, they always corresponded with numbers with zeros. In the grammatical condition, each stimulus consisted of two grammatical segments with 2–4 words in each, corresponding either with two 4-digit numbers or with a 4-digit number and a 5-digit number. Each of these numbers included at least one 0. In the fragmented condition, the words of each stimulus were reshuffled into segments as short as possible—only the word “thousand” could be merged with the preceding/next word. Thus, in the fragmented condition each segment included 1–3 words, and no stimulus included more than one multi-word segment. The two conditions were administered as two separate blocks, in counterbalanced order, with 20 trials in each block.

The data coding and the statistical analysis were as in Experiment 1. However, the word “thousand” was classified differently. Experiments 1–3 included only 5- and 6-digit numbers, and in spoken Hebrew, the word “thousand” in these numbers is a separate word, similar to the multiplier word “thousand” in English. Consequently, this word was classified as a single morpheme—a lexical class without a digit. In contrast, the present experiment also included 4-digit numbers. In spoken Hebrew, in these numbers the thousands word is a single word with two morphemes, similar to the hundreds words. For example, the Hebrew word for 3 is /shalosh/, “thousands” is /alafim/, but 3000 is not a simple concatenation of these two words, it is /shloshtalafim/. Thus, in 5- and 6-digit numbers, we considered the word “thousand” like in Experiments 1–3—as a single morpheme, a class without a digit; but in 4-digit numbers, we considered each thousands word as two morphemes—a class (“thousand”) and a digit.

### Results and discussion

The results were similar to the previous experiments: The morpheme error rate, digit error rate, and class error rate were lower in the grammatical condition than in the fragmented condition (Fig. 6b; with the LMM described in Experiment 1 “Statistical analysis” section, morphemes: Δ = 10.6%, *χ*^2^ = 128.0, *p* < 0.001; digits: Δ = 10.4%, *χ*^2^ = 94.6, *p* < 0.001; classes: Δ = 10.8%, *χ*^2^ = 124.1, *p* < 0.001). The morpheme error rate was lower in the grammatical condition than in the fragmented condition even for each single participant (Additional file 1: Fig. S3). Namely, the participants managed to capitalize on the number syntax as the basis for chunking even when the syntactic structure was irregular, and even when it changed on each trial. The effect sizes (difference in error rates between the grammatical and fragmented conditions) were similar here and in Experiment 3, which had a similar design but used only regular numbers without 0 and 1 (morphemes: *t*(38) = 0.38, one-tailed *p* = 0.26; digits: *t*(38) = 1.36, one-tailed *p* = 0.09; classes: *t*(38) = 0.003, one-tailed *p* = 0.50). This suggests that representing irregular and dynamic syntactic structures was as easy as representing regular, fixed structures.

## Experiment 5: a cross-triplet representation of syntax

A critical finding in Experiment 1 was that in condition A, in which the stimuli were grammatical 6-digit numbers, the performance was worse than in condition B, in which each stimulus was a pair of 3-digit numbers. We concluded that the syntactic representation of numbers is not limited to 3-digit numbers—it can also capture longer numbers, with 5 or 6 digits. In condition A, this large-number representation caused the creation of oversized chunks, which were hard to remember.

Experiment 5 aimed to provide additional support to this conclusion. To this end, we used stimuli similar to Experiment 1’s conditions A and B, but now the numbers included the digit 0, so there were fewer words in each stimulus. The idea was simple: In Experiment 1, the greater chunking in condition A led to poorer performance because the participants created mega-chunks that exceeded their working memory capacity. Here, the numbers contained fewer words (because they included 0), so even in the grammatical condition each grammatical segment could fit in the participants’ working memory capacity. We predicted that in this experimental setting, syntactic chunking would lead to better performance in the grammatical condition than in the fragmented condition, a pattern opposite to Experiment 1.

### Method

The participants were 30 adults aged 19;4–38;3 (mean = 27;1 SD = 4;9). The design was similar to Experiment 3 (overt cueing), with two conditions—grammatical and fragmented. Each stimulus was based on two 5- and 6-digit numbers, each containing 2 or 3 zero digits and 3 non-zero digits, such that each of the two numbers had 4 words—the word “thousand” and 3 other words (totaling 8 words per stimulus). In the grammatical condition, the stimulus was the two number names (e.g., *thirty thousand fourhundred five; sixhundred thousand seventy nine*). In the fragmented condition, each of the two numbers was broken into two grammatical segments by moving the word “thousand” to be last (*thirty fourhundred five thousand*).^1^ Importantly, the grammaticality manipulation did not affect any within-triplet syntactic relations, and both conditions used precisely the same words. Thus, the only difference between the conditions was in whether each pair of triplets was connected into a single grammatical segment or not. The two conditions were administered as two separate blocks, with 20 trials in each block. The order of blocks was counterbalanced across participants. The data coding and the statistical analysis were as in Experiment 1.

### Results and discussion

**Fig. 7:**
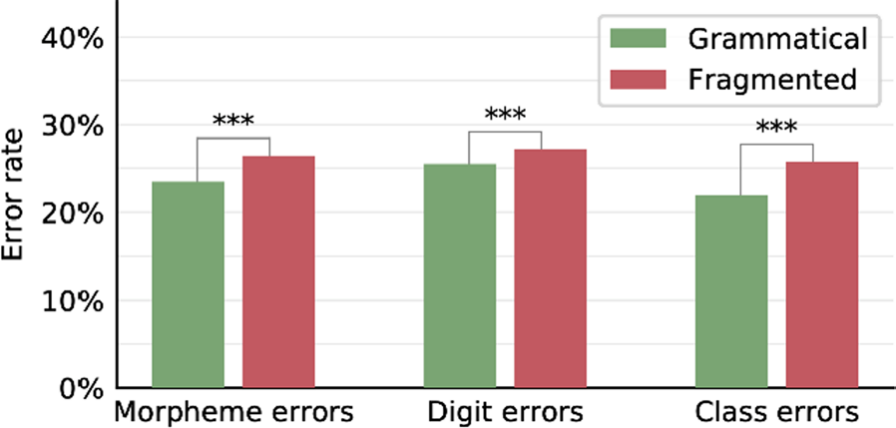
Results of Experiment 5. In the grammatical condition, each pair of triplets was merged by the word “thousand.” In the fragmented condition, the word “thousand” was misplaced at the end of each stimulus (number-word sequence). Supporting the idea of a cross-triplet representation, i.e., that the number’s syntactic representation can merge words from two different triplets, the morpheme error rate, digit error rate, and class error rate were lower in the grammatical condition than in the fragmented condition. Asterisks denote the significance of the Condition factor in the linear mixed model described in the text (all *p* < .001)

The morpheme error rate, digit error rate, and class error rate were lower in the grammatical condition than in the fragmented condition (Fig. 7; with the LMM described in Experiment 1 “Statistical analysis” section, digits: Δ = 1.9%, *χ*^2^ = 7.87, *p* = 0.005; classes: Δ = 3.6%, *χ*^2^ = 27.7, *p* < 0.001. The morphemes LMM did not converge so we ran it without the Stimulus random factor: Δ = 3.0%, *χ*^2^ = 20.4, *p* < 0.001. Per-participant results in Additional file 1: Fig. S4). Namely, the participants were sensitive to the syntactic difference between the two conditions: In the grammatical condition, they merged the two triplets into a single chunk, whereas in the fragmented condition they did not, or they did so less often. These results indicate that the participants represented the number’s cross-triplet syntactic structure.

The opposite patterns observed here and in Experiment 1 strengthen the conclusion that the participants created a cross-triplet syntactic representation. When the cross-triplet representation, which turned into a single chunk, included sufficiently few words to fit in the participants’ working memory capacity, the syntactic chunking improved memorization. This was the case here. When the cross-triplet representation contained too many words, as in condition A in Experiment 1, the same chunking process resulted in oversized chunks that disrupted memorization.

## General discussion

### The syntactic representation of multi-digit numbers

This study examined the core syntactic representation of multi-digit numbers using a syntactic chunking paradigm—repetition on grammatical versus fragmented sequences of number words. We reached several conclusions

#### 1. A core representation of the syntactic structure of numbers

Our basic finding was clear and robust across 5 experiments: The participants remembered grammatical sequences of number words better than non-grammatical ones. This finding is hard to explain as resulting from a low-level syntactic mechanism such as those reviewed in the Introduction—e.g., detecting the order of words and the decimal class of each word, or retrieving the number’s morphological affixes during verbal production. The better explanation is that the participants created a syntactic representation of the number in each grammatical segment, and this allowed representing the words of each segment as a chunk in working memory. This chunking, in turn, improved memorization.

The finding of a core cognitive representation of the number syntax is a conceptual replication of Dotan et al. (2021a). As we shall now see, our findings also extend Dotan et al.’s conclusions in several ways.

#### 2. A whole-number syntactic representation, not just a pairwise merge

Hung et al. (2015) postulated a syntactic process that merges adjacent pairs of number words into a single syntactic structure when the two words are grammatically mergeable. An important question is whether our findings can be explained by such a pairwise merge, i.e., by a local syntactic operation, without assuming the whole-number syntactic representation. Such a pairwise merge can easily explain the advantage of a particular grammatical condition over a fragmented one. It can even explain a monotonous increase in performance for increasingly longer grammatical segments, as we observed in Experiment 1 conditions D, C, B—for example, longer grammatical segments may increase the likelihood for a pairwise merge to occur. Critically, however, a pairwise merge cannot explain the discontinuity we observed in Experiment 1—the finding that performance deteriorated beyond a certain grammatical segment length. This finding therefore refutes the interpretation of our data as merely reflecting a pairwise merge. In contrast, the whole-number representation view can readily account for this discontinuity. Grammatical segments can be represented as a single whole-number syntactic structure; when the segment is too long, it becomes an oversized chunk in working memory, which is hard to remember.

Importantly, although our findings cannot be interpreted by a local merge without assuming a whole-number representation, a plausible assumption is that a pairwise merge operation, which is applied recursively, serves as the foundation for the full-fledged hierarchical syntactic representation of the whole number. In fact, this is precisely what Hung et al. (2015) hypothesized. We return to this point in later below section.

#### 3. Compressibility versus detectability

Experiments 2–4 showed that the syntactic chunking effect cannot be explained as an artifact of grammatical segment detectability, i.e., the putative easier detection of long grammatical segments relative to short segments. While our experimental manipulation may have affected detectability, such an effect does not suffice to account for our findings: Controlling for detectability did not eliminate or even reduce the syntactic chunking effect (Experiment 3); and a strong and similar-sized syntactic chunking effect was observed even when the syntactic structure was changed on each trial (Experiments 2, 4), a situation that should disrupt detectability. Thus, the best explanation of the syntactic chunking effect is not that the grammatical segments are more detectable, but that they are more compressible because they induce a syntactic representation.

#### 4. The syntactic structure is created dynamically, by a generative process

A critical finding in Experiment 1 was that the best performance was not in condition A, which had the longest grammatical segments, but in condition B, which created an optimal balance between the segment length and the number of segments. We concluded that a syntactic representation was created even for the long segments in condition A, but the ability to maintain the resulting chunks in memory was limited by working memory capacity, such that oversized chunks disrupted performance. This pattern, in which the ability to remember a chunk is capped by working memory capacity, is not typical to chunks based on a predefined rigid template, as such templates should not be subject to the working memory capacity limit (Cowan, 2001). Rather, the pattern fits a situation in which chunks are created ad hoc and dynamically, i.e., the specific chunk structure is recreated on each presentation of a new number. In our case, this dynamic ad hoc chunking process is syntactic.

#### 5. A cross-triplet syntactic representation

When our stimuli included very long grammatical segments, the pattern of results depended on how the long segment loaded working memory: In Experiment 1, the long grammatical segments had many words, and the resulting chunks were hard to maintain in working memory, so accuracy dropped. In Experiment 5, because the numbers had zeros, the long grammatical segments did not include that many words, so they could fit within the participants’ working memory capacity limit, and the syntactic chunking improved memorization. This pattern demonstrates that the participants created a syntactic representation even for these long segments, which were based on 5- or 6-digit numbers. Namely, the syntactic representation is not limited to a single triplet, in which the syntactic structure is relatively simple (in Hebrew 3-digit numbers, each word has a different class). Rather, the syntactic representation can capture 5- and 6-digit numbers, with their cross-triplet structure and inner hierarchy, as should be expected from a hierarchical syntactic representation.

#### 6. A syntactic representation for both regular and irregular numbers

A syntactic representation can be created for regular numbers, which include only the digits 2–9 (Experiments 1, 2, 3). It can also be created for irregular numbers (Experiments 4, 5)—numbers that include the digit 0, which causes a “missing word” in the verbal numbers, and numbers with teen words, which violate the regular decade-unit structure. In fact, the syntactic chunking effect seemed as strong for irregular numbers as it was for regular numbers. At least for adults without learning disorders, it seems that creating the more-complicated syntactic structures is not considerably harder than creating the simple structures. However, the situation may be different for individuals with cognitive deficits, e.g., if they have a syntactic disorder (Dotan & Friedmann, 2018), as well as for children who did not yet acquire syntactic proficiency (Power & Dal Martello, 1990, 1997; Shalit & Dotan, 2022).

Taken together, the conclusions that the core syntactic structure can be created for regular and irregular numbers, and that it can be created across triplets, mean that literate adults can represent the syntactic structure of natural numbers at least up to 1,000,000.

#### 7. Automatic processing of the number syntax

The participants processed the number syntax, and used it to chunk number words, not only when this was beneficial but also when the chunking disrupted memorization (Experiment 1). This finding suggests that the creation of a syntactic representation was not a voluntary, conscious-strategic decision, but an automatic process. It also strengthens the idea that our findings imply on a genuine cognitive representation of syntax and do not reflect some grammar-based overt strategy.

#### 8. Language

We showed syntactic chunking in Hebrew, a Semitic language. Previous studies showed number-grammaticality effects in French, a Latin language, and in Chinese, a Sinitic language (Barrouillet et al., 2010; Hung et al., 2015). These three languages have very different morpho-syntactic characteristics, not only for the language as a whole but also specifically in the verbal number system. Although these three studies tap slightly different syntactic processes (as we detail below), the existence of a grammaticality effect in three languages so different shows the robustness of the sensitivity to number syntax. This is a global phenomenon, not the result of particularities of a specific language or a specific syntactic system.

### Syntax as the “glue” of lexical items

Across our 5 experiments, similar result patterns were observed in all 3 measures that we used: the class error rate, the digit error rate, and the morpheme error rate. Arguably, each of these measures taps a slightly different aspect of cognitive processing. The class error rate taps number syntax in the most direct manner, because the number words’ classes reflect the syntactic structure of verbal number (the so-called number-word frame, Cohen & Dehaene, 1991; Dotan & Friedmann, 2018). In contrast, the digit error rate reflects only the digit values, so it is orthogonal to the number’s syntactic structure; it does not distinguish between words such as “four,” “forty,” and “fourhundred.” The finding of a syntactic chunking effect in the digit error rate, although this measure does not tap syntax directly, suggests that the memorization of digits depends on the memorization of syntax. A simple explanation of this finding is that the syntactic structure is not “pure” and independent, but rather the digit values are embedded in it. Future studies may look more deeply into how the digit values are integrated with the number’s syntactic structure.

### Previous investigations of syntactic chunking

Two previous studies used an experimental manipulation similar to ours—presenting number-word sequences with varying lengths of grammatical segments (Barrouillet et al., 2010; Hung et al., 2015). Both studies showed that the participants processed the number syntax, and in this sense, they are similar to the present study. Nevertheless, the three studies are also different from each other in critical respects, and they complement each other, because they examined 3 different syntactic abilities.

Barrouillet et al. (2010) asked 5-year-old children to repeat grammatical or fragmented sequences of number words and showed better memorization of the grammatical sequences than of the fragmented ones—a syntactic chunking effect. Namely, the children clearly exhibited syntactic knowledge. However, whereas our study examined the core cognitive representation of number syntax, Barrouillet et al. probably examined different syntactic abilities. Indeed, given the young age of their participants (pre-school), Barrouillet et al. did not interpret their own findings in terms of a whole-number syntactic representation as we did, but proposed that the children used partial syntactic knowledge about the verbal number system. Such knowledge is dissociable from the ability to actually use syntax when saying numbers (Shalit & Dotan, 2022). Thus, unlike our study, Barrouillet et al. cannot inform about the core representation of syntax. At the same time, this study informs about early knowledge of the syntactic system of numbers—an important aspect of number syntax, which we could not examine with our adult participants.

Hung et al. (2015) asked literate adults to read aloud sequences of number words in several degrees of grammaticality (similar to our Experiments 1 and 2), with the words presented on screen one at a time. Sequences with longer grammatical segments were read faster and induced higher activity in the left inferior frontal gyrus and in the left inferior parietal lobe. These findings clearly show that the participants processed the numbers’ syntactic structure; however, they do not necessarily show the existence of a core, whole-number syntactic representation. Indeed, Hung et al. did not interpret their own findings in terms of whole-number syntax but in terms of binary merge—a local syntactic operation that merges two adjacent words when they grammatically match each other. In contrast, our findings clearly indicate a core syntactic representation of the whole number. In this sense, the two studies complement each other: Hung et al. showed that number words are merged by a binary syntactic operation that combines adjacent words, and our study showed that this merge is not merely a local effect, but serves as the foundation for a whole-number syntactic representation (similar to the role of the merge operation in the syntax of sentences, Chomsky, 1995)—precisely as Hung et al. hypothesized.

An interesting possibility is that our paradigm and Hung et al.’s (2015) paradigm systematically tap different levels of syntactic processing: Our paradigm taps a core, whole-number syntactic representation, whereas Hung et al.’s taps a more fundamental syntactic operation—the binary merge. Several factors suggest that this might indeed be the case. First, Hung et al. asked their participants to read aloud sequentially presented number words, a task that seems to encourage word-by-word processing, whereas we used a memorization task, which seems to encourage a whole-number processing. Second, Hung et al. measured the reaction time for each word, which may tap a local effect, and the brain activity measured in fMRI, which may be additive across several local merge operations due to the low temporal resolution of the BOLD signal. In contrast, we measured the degree of memorization, which is presumably affected by the load imposed on working memory by the whole number. Third, while the findings of the two studies were generally consistent with each other, they nevertheless diverged in a critical condition—the very long grammatical segments. Hung et al. showed monotonous improvement (faster responses, higher brain activity) even in this condition, as should be expected when examining the additive effect of several local syntactic-merge effects, whereas our data showed a U-shaped pattern with optimal performance in a mid-sized segment length, as should be expected when examining the memorization of a whole-number representation. All these arguments support the idea that our paradigm and Hung et al.’s may tap different levels of syntactic representation. Future studies may use both paradigms, perhaps even in conjunction, to examine fine-grained aspects of the syntactic representation of numbers.

Importantly, our findings extend these two previous studies in several ways, detailed in the previous sections. We showed a core representation of a whole-number syntax; that syntactic chunking results from the compressibility of syntactic segments rather than from their detectability; that the syntactic representation is created via a dynamic, generative process, rather than retrieved as a predefined template; that it is not limited to short numbers or to simple syntactic structures; that its creation is automatic rather than voluntary; and that it exists in a Semitic language.

### A hierarchical representation of number syntax

Our data show that literate adults represent the syntactic structure of numbers. An interesting question is whether this syntactic representation is hierarchical, as proposed by Michael McCloskey and his colleagues (McCloskey, 1992; McCloskey et al., 1986), similar to the syntactic representation of sentences in natural language (Chomsky, 1956). Several aspects of our data agree with the notion of a hierarchical representation of number syntax. First, we showed that adults can represent the syntactic structure also for long numbers (with 5 and 6 digits). This is important because if the syntactic representation was limited to shorter numbers, any hierarchy would have been minimal/trivial. Second, we showed that the syntactic representation can include more elements (words) than the working memory capacity limit of 3–4 words. This is exactly what we should expect from a hierarchical representation. Arguably, one advantage of hierarchical representations is precisely their ability to transcend the size of a single working memory chunk. Third, when syntactic segments were long (more than 3–4 words), they were not represented as a template-based mega-chunk; rather, they were created by a generative process, and the creation of hierarchy is presumably such.

We acknowledge that these findings, while in agreement with the notion of hierarchy, still do not strictly prove that the syntactic representation is hierarchical. Additional evidence for hierarchy may come from other methods. For example, an ongoing study in our laboratory (Barash & Dotan, 2019) used a number-dictation task and showed that the time gaps between adjacent digits follow a tree-like hierarchical structure, thereby indicating more directly that the underlying syntactic representation is hierarchical.

### Pedagogical implications

Our findings have several pedagogical implications with respect to potential sources of difficulty in number processing, including learning disorders, and with respect to best-practices for teaching children how to read and write numbers.

First, we showed that the syntactic representation is created by a generative process, not by retrieving predefined templates. Consequently, one may hypothesize that to teach number syntax, we should focus on teaching the generative syntactic rules rather than rehearse specific syntactic structures or even specific numbers. In accord with this idea, Power and Dal Martello (1990, 1997) showed that some children make mistakes in specific syntactic rules. Another study (Shalit & Dotan, 2022) further suggests that children learn each syntactic rule explicitly and do not necessarily generalize from one syntactic rule to another; and that they don’t even always learn the rules for smaller numbers before the rules for larger numbers. This idea—that we should teach the syntactic rules of numbers—appears to be simple, yet in many schooling systems it is not implemented. Very often, children are not taught these rules explicitly, but rather we assume that they will grasp the rules implicitly via exposure to numbers. Although this implicit learning eventually works for most children, it seems to require a lot of effort (Cheung & Ansari, 2020).

Second, the syntactic representation handles even relatively large numbers, at least up to 1,000,000. Thus, it seems we should teach and train the syntactic rules also for these large numbers, not only for small numbers (on which schooling typically focuses). This may perhaps be particularly helpful for those with difficulties or dysnumeria (a number-reading learning disorder), who seem to struggle specifically with the larger numbers (Dotan & Friedmann, 2018).

Third, the syntactic structure is created automatically rather than voluntarily, so it makes sense to train it as we train automatic processes. For example, a plausible hypothesis is that to develop children’s ability to read and write numbers, we should focus on training and practice of the syntactic rules, rather than teach only the conceptual aspects of the decimal system. At present, this is not what happens at school: At least in some countries, elementary-school curriculum specifically addresses the issue of syntax and base-10 structure at the conceptual level, but does not focus on automatizing the digits-to-words and words-to-digits conversion.

Fourth, our findings join a growing number of studies, which together show that “number syntax” is not a single cognitive process but a combination of several different processes and representations: There are several “low level” mechanisms that handle specific types of syntactic information (Cohen & Dehaene, 1991; Cohen et al., 1997; Dotan & Dehaene, 2020; Dotan & Friedmann, 2018, 2019; Dotan et al., 2021b; Furumoto, 2006; Hayek et al., 2020; Kallai & Tzelgov, 2012; Lambert & Moeller, 2019; McCloskey et al., 1986; Zuber et al., 2009), and there is a core syntactic representation of the whole number (the present study and Dotan et al., 2021a), which seems to be based on a pairwise merge operation (Hung et al., 2015). As we come to understand these mechanisms in greater detail, we learn about the different types of dysnumeria—the family of learning disorders that result from a deficit in each of these mechanisms (Dotan & Friedmann, 2018): We learn how to create methods to identify the different types of dysnumeria and to treat them.

Last, it seems that our paradigm, which was initially developed as an experimental tool to study number syntax, stands a chance to be useful also pedagogically. In the present study, most of the experiments showed significant results not only at the group level but also for each participant; this suggests that syntactic chunking may be sensitive enough to be used as a diagnostic method to detect, for single individuals, deficits in the representation of number syntax. A possible prediction is that a person with impaired syntactic representation may be unable to represent large syntactic structure and would therefore show abnormal syntactic chunking patterns in the conditions with long grammatical segments. Moreover, the syntactic chunking paradigm may perhaps be used also to improve syntactic abilities: The comparison of our data with Hung et al.’s (2015) stresses the advantage of the number-memorization paradigm to target the whole-number syntactic representation. A possible prediction is that to treat an impaired syntactic representation, it may be advantageous to not only expose the person to grammatically valid numbers, but also require the person to memorize them.

## Conclusion

An increasing body of research shows that the syntactic structure of numbers is handled by a multitude of cognitive processes. Most of these processes seem to handle highly specific, low-level syntactic aspects of the number. Our present data join a few studies indicating that on top of these low-level processes, there exists a core syntactic representation of the whole number. We showed several specific characteristics of this representation, in particular that it is cross-triplet, generative, and automatic in the sense that it does not seem to arise from a voluntary decision—precisely the characteristics we should expect from a hierarchical representation. Future studies may examine more deeply whether the representation of numbers is indeed hierarchical, as hypothesized by McCloskey and colleagues more than 3 decades ago, and provide additional insight as to how humans (and why only humans) speak the language of mathematics.

Our findings also have several possible pedagogical implications. In particular, we proposed that it might be important to teach not only the conceptual and mathematical aspects of the base-10 systems, but also the specific ability to read and write numbers; that it may be better to teach the rules of number syntax rather than merely use examples and implicit learning; and that these rules should be taught and trained also for large numbers, not only for small numbers. Finally, we proposed that the syntactic chunking method may potentially be useful for assessment and treatment of learning disorders related to number-syntax representation. Future studies may examine these hypotheses in pedagogical settings, hopefully paving the way to better educational methods for teaching numerical literacy and addressing the related learning disorders.

## Supplementary Information

**Additional file 1: **Supplementary figures.

## Data Availability

The datasets generated and/or analyzed during the current study are available in the OSF repository http://osf.io/86kjz. No experiment was pre-registered.

## Footnotes

1 Note that the participants can only interpret this sequence as the two numbers 30 and 405,000. They cannot interpret this word sequence as denoting “3405 thousand”: In spoken Hebrew, “four-hundred” counts as a single word, so “thirty four-hundred” unambiguously means 30 and 400.
