# Correction: Syntactic chunking reveals a core syntactic representation of multi-digit numbers, which is generative and automatic

**DOI:** 10.1186/s41235-022-00426-1

**Published:** 2022-07-31

**Authors:** Dror Dotan, Nadin Brutmann

**Affiliations:** Mathematical Thinking Lab, School of Education and School of Neuroscience, Tel Aviv University, Tel Aviv, Israel

## Correction to: Cognitive Research: Principles and Implications (2022) 7:58 10.1186/s41235-022-00409-2

The original article Dotan and Brutman (2022) contained an error in co-author, Nadin Brutmann’s name which has since been corrected.
