# Supplementary material for: Syntactic chunking reveals a core syntactic representation of multi-digit numbers, which is generative and automatic
Source: Cogn Res Princ Implic. 2022 Jul 6;7:58. doi: 10.1186/s41235-022-00409-2 (PMC9259776; doi:10.1186/s41235-022-00409-2)
Supplement: Supplementary file 1 — Additional file 1: Supplementary figures. [file 41235_2022_409_MOESM1_ESM.pdf]

Supplementary material for

Syntactic chunking reveals a core syntactic representation of multi-digit numbers, which is generative and automatic

Dror Dotan and Nadin Brutman

Syntactic chunking effect per participant

Figures S1, S2, S3, and S4 show the syntactic chunking effect for each participant. In these figures, the participants are sorted according to the effect size: Δ(morpheme error rate) between the grammatical and fragmented conditions. Experiment 2 was the only experiment in which the syntactic chunking pattern was not observed for each participant. This is perhaps not surprising, given that this experiment had the weakest statistical design, for two reasons: (1) the number of items was small – 20 items for 3 conditions altogether, rather than 20 items per condition. (2) The items were different in each condition, so the statistical analysis was between items and not within item.

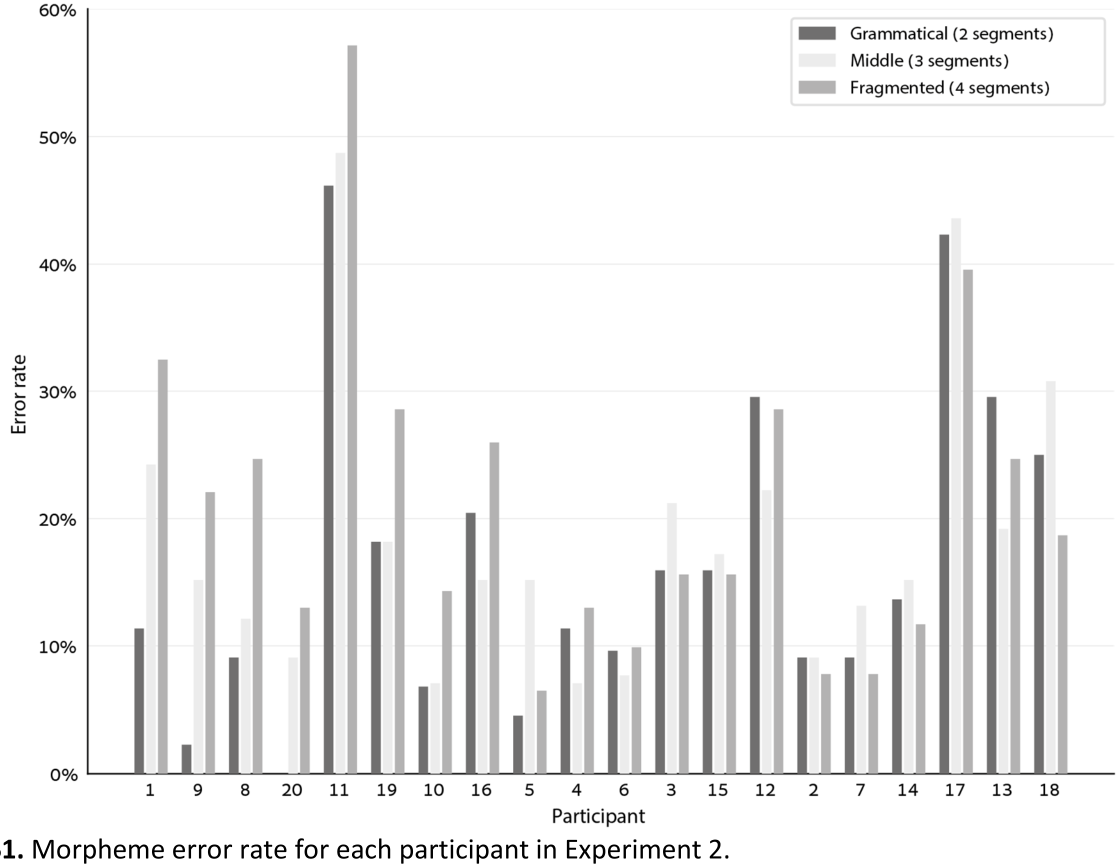

Fig. S1. Morpheme error rate for each participant in Experiment 2.

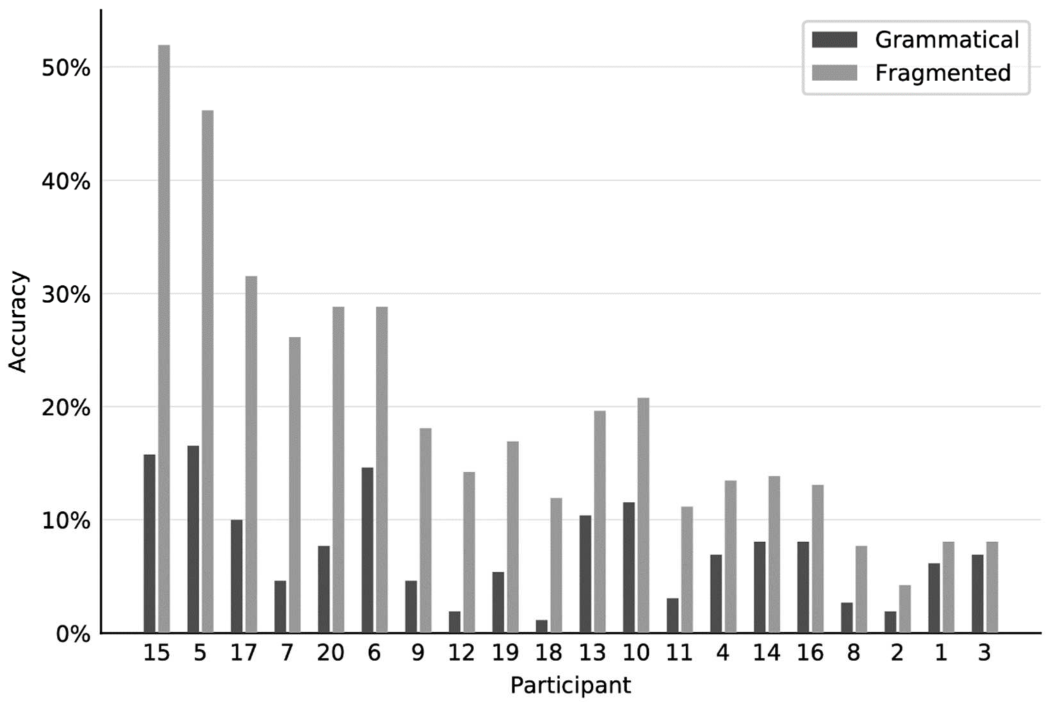

Fig. S2. Morpheme error rate for each participant in Experiment 3.

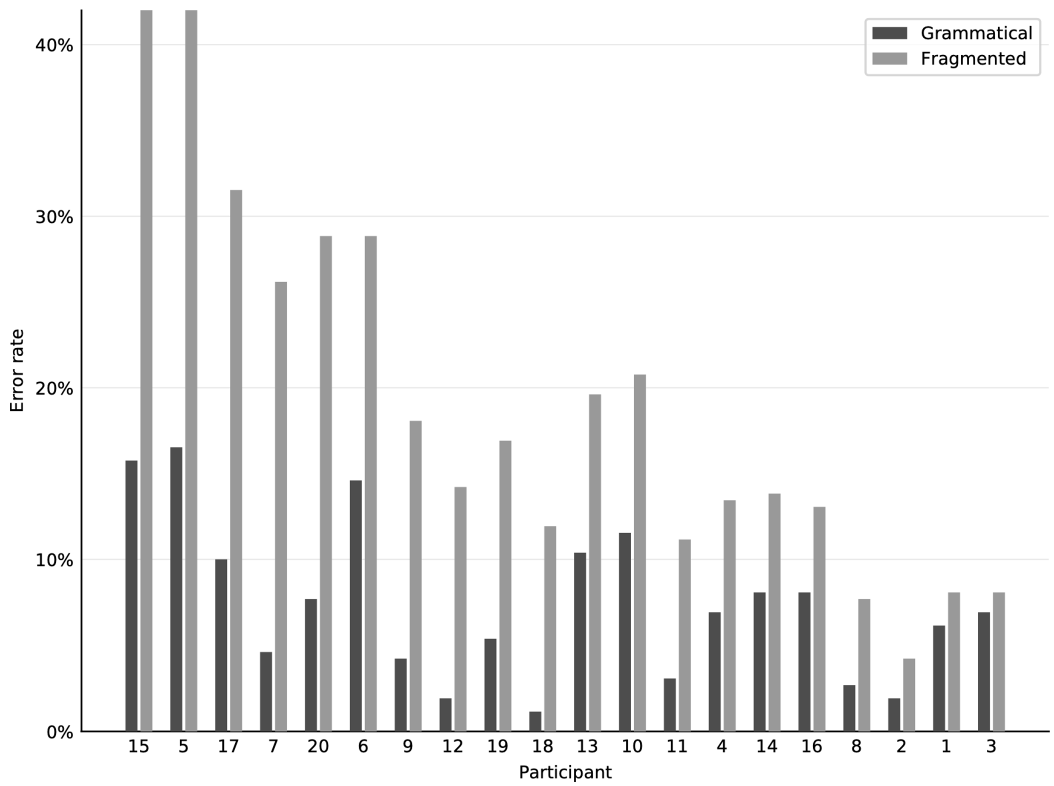

Fig. S3. Morpheme error rate for each participant in Experiment 4.

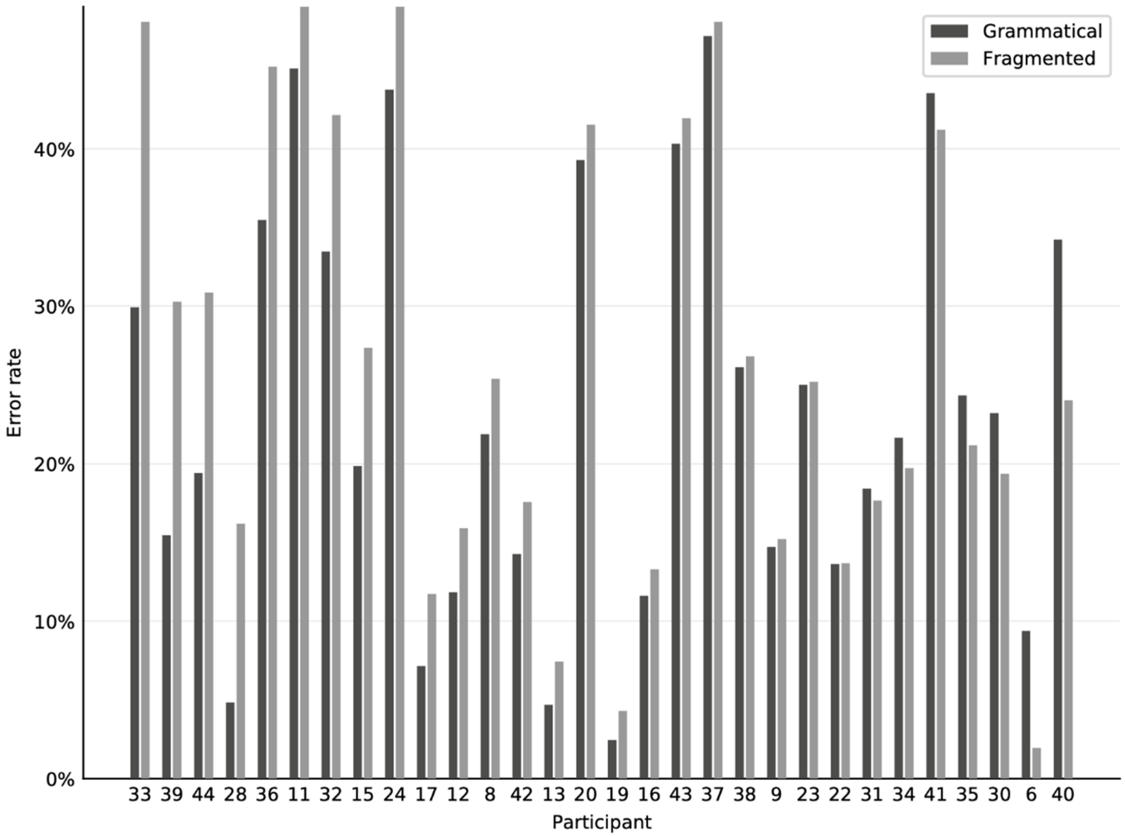

Fig. S4. Morpheme error rate for each participant in Experiment 5.
